# Minimalism in the Light of Biology: What to Retain and What to Discard?

**DOI:** 10.3389/fpsyg.2019.01303

**Published:** 2019-06-04

**Authors:** Ljiljana Progovac

**Affiliations:** Linguistics Program, Department of English, Wayne State University, Detroit, MI, United States

**Keywords:** minimalism, language evolution, move, subjacency, recursion

This volume, and in general this moment in the history of science, is calling for us linguists, and especially those of us who have worked in Minimalism, to characterize what it is that our approach has discovered, that we want to embrace and move forward with, and what it is that we need to discard. There is plenty in both categories, and it is precisely the considerations of biology (e.g., language evolution) that can help us weed out the burdensome, damaging aspects of this approach. Too often we linguists look down upon the study of language evolution as some kind of marginal topic that need not concern “true” linguists, and we prefer to just wait until geneticists, biologists, and neuroscientists figure it all out. And yet, it is only linguists who can put forward specific, linguistically informed hypotheses that can be subjected to interdisciplinary testing. The emphasis here is on specific, falsifiable hypotheses, rather than some vague assertions that cannot be subjected to falsification. It is true that many such specific hypotheses will be proven wrong, but after all, the nature of the scientific process is simply to narrow down the range of possibilities.

My focus here is on a few influential assumptions/postulates in Minimalism that are particularly harmful in establishing meaningful links between language and biology, and which, both on this ground, and based on more careful linguistic considerations, should be abandoned. I will also point to certain postulates that are worth keeping and moving forward with, based on their usefulness for biological considerations. Needless to say, this short Opinion piece is not a comprehensive review of Minimalism, but is rather meant to provoke a substantive discussion about how to better constrain this framework, and how to, at the same time, make it better compatible with gradualist evolutionary considerations.

## Omni-Potent, Infinite Merge

Consider the recent claims, primarily in the context of language evolution research, that syntax reduces to Merge (e.g., Berwick and Chomsky, 2011, 2016), where Merge has been granted almost mythical powers, distracting from the fact that Merge is really just a term meaning “combine.” It is true that we can always pack into such terms whatever we want, e.g., that it has to be binary; that it is recursive; that it subsumes Move. But these are separate and separable properties of human syntax, and need not be seen as the part and parcel of Merge at all. Packing virtually all we know about syntax into Merge is only there to create an illusion that syntax is one undecomposable, unnegotiable block, which evolved as a result of one single sudden evolutionary event, as per Berwick and Chomsky's saltationist view. The binary nature of syntactic structure building (e.g., Kayne, 1984), as well as the small clause foundation of every sentence (section Binarity and Hierarchy of Projections), are stable postulates, in fact discoveries, which predate Minimalism, as well as carry over into Minimalism. Because they are also useful for evolutionary considerations, they are postulates to keep and move forward with. On the other hand, the claims that Merge by itself yields infinite recursion, and that it subsumes Move, are problematic both from the standpoint of linguistic analysis, and language evolution considerations. I discuss Move in section Subjacency, and infinite recursion in section The Notion of Free Infinite Recursion. Section Syntactic Uniformity Across Languages and Constructions discusses the idea of syntactic uniformity across languages.

## Subjacency

In Minimalism, Move is typically seen as a default state of grammar (in fact, an inseparable property of Merge itself), in the sense that it applies freely and repeatedly, as long as there are features to check. With that assumption, one of the central goals of syntactic theorizing has been to provide an elegant characterization of Subjacency, the principle supposed to explain why Move is nonetheless prohibited from some syntactic constructions, dubbed islands. The expectation is that there exists some deep, abstract property which captures islandhood effects in a unified and elegant fashion (but see e.g., Cinque, 1978; Postal, 1997; Boeckx and Grohmann, 2007; Boeckx, 2008; Sprouse and Hornstein, 2013 for an exploration of alternative views).

Typically, Move is possible only out of (a subset of) complements/objects, while the constructions that prohibit Move are many and various, including adjuncts, conjuncts, subjects, complex noun phrases, *wh*-clauses (for a long list of additional islands, see Postal, 1997, 1998). Crucially, constructions which prohibit Move (islands) do not form a natural class, while those that allow Move do form a natural class (Progovac, 2009, 2015). It is thus not surprising that, despite the sustained effort for half a century (since Ross, 1967), to date there has been no unified or principled account of islandhood (Belletti and Rizzi, 2000 report an interview with Chomsky, in which he concludes that much). The classic accounts are Huang (1982); Lasnik and Saito (1984); Chomsky (1986), but there have been many more attempts, including the so-called phases in Minimalism (Chomsky, 2001).

If islands do not form a natural class (i.e., have no thread in common), then there cannot possibly exist an insightful unified account of islandhood. Accordingly, our efforts need to be re-oriented from searching for a common thread for all islandhood to searching for an explanation for what accommodates Move in non-islands, and why islands exist at all. This shift in direction would argue against the reduction of Move to (internal) Merge, given that (external) Merge is not affected by islandhood, but Move is. There is in fact a good evolutionary rationale for why islandhood (i.e., lack of Move) should be the default (evolutionarily primary) state of grammar (Progovac, 2009, 2015), while there is no good explanation for why evolution would deliver a principle such as Subjacency, against the background of free and powerful Move (e.g., Lightfoot, 1991). It is significant that the elusiveness of Subjacency has been used to argue that syntax could not have evolved gradually: one does not see why evolution would target a grammar with Subjacency, when its contribution to grammar is not clear, let alone its contribution to survival. According to Lightfoot (1991), “subjacency has many virtues, but … it could not have increased the chances of having fruitful sex.” Subjacency thus poses (unsolvable) problems not only for syntactic theory, but also for language evolution! These are two important reasons to abandon Subjacency, and the concomitant assumptions.

This search for an abstract principle accounting for all islandhood is analogous to biologists seeking a unified, deep biological explanation for why so many species do *not* have wings. However, instead of assuming that every living being is supposed to have wings by default, biologists consider that having wings is an evolutionary innovation, against the backdrop of the primary state of winglessness. Applying this logic to syntax, one would need to posit that there existed a modest proto-syntax stage without wings (i.e., without Move, recursion, and many other niceties of syntax), and that Move was a later innovation, enabled by the rise of hierarchical syntax (Progovac, 2009, 2015). As is well motivated in syntactic theory, Move typically requires hierarchical syntax and c-command.

## The Notion of Free Infinite Recursion

There are many constructions across languages that are not (infinitely) recursive, i.e., do not allow multiple embeddings of one category within another category of the same type, even though they are presumably put together by Merge^1^. These include recursive embedding of clauses (CPs) within other clauses, or possessive noun phrases (NPs/DPs) within other noun phrases, in some languages, as well as recursive composition of noun-noun compounds in some languages. There are even rigid syntactic creations (often small clauses) across languages, including English, that are syntactic isolates in the sense that they do not merge/combine with any category, and not just with categories of the same type. These are all discussed and exemplified in Progovac (2015, and references there), adducing evidence that recursion, Merge, and Move are decomposable into primitives and simpler constructs. What leads to recursive possibilities in some constructions, in some languages, is a combination of factors, including not only Merge, but also specialized functional projections and categories such as complementizers, which develop/evolve through gradual grammaticalization processes (e.g., Heine and Kuteva, 2007). In other words, the reason why languages (in some constructions) show recursion (and potential infinity) need not come from some cognitive twist in the brain that suddenly emerged in humans, but rather from a complex interaction of (grammatical) factors, some of which are not available in all constructions, in all languages.^2^

## Syntactic Uniformity Across Languages and Constructions

Finally, this brings up yet another undesirable postulate, promoted by some, but certainly not all syntacticians, that the syntax of every construction is syntactically uniform across all languages. Whatever functional projections and categories have been postulated for English sentences (e.g., vP, TP, CP, DP, DegP) must also be posited in equivalent constructions/translations in all the other languages. How one translates these languages into English is how one can analyze them syntactically. When something looks different from the English situation, one can simply posit some null category or another, ironing out the differences. While in some cases one may be justified in positing a null category, this blanket declaration of uniformity takes away the tools necessary for deciding when this is justified, and when not, giving rise to a host of unfalsifiable claims. Such sweeping claims are also harmful for biological considerations, because it is exactly the variability, the contrasts and comparisons among different constructions, that reveal the evolutionary primitives of syntax (e.g., Progovac, 2015, 2016), which can also be used to probe language variation and language representation in the brain (e.g., Progovac et al., 2018a,b).

## Binarity and Hierarchy of Projections

At the same time, the basic analysis of the sentence in this framework (Minimalism and predecessors), in English and related languages, is quite insightful and useful for both linguistic and biological evolutionary considerations. Every sentence in this framework is analyzed as having as its bottom layer a Verb Phrase (VP) or Small Clause (SC), essentially a mini sentence, upon which other layers can be constructed, in a binary fashion, including: vP, another (transitive) layer of VP; TP, Tense Phrase; and CP, Complementizer Phrase.

(1) $$\begin{array}{l} CP>TP>vP>SC/VP \end{array}$$

Binary branching and the small clause foundation are amongst the most insightful and stable postulates in this theoretical framework (e.g., Burzio, 1981; Stowell, 1983; Kitagawa, 1985, 1986; Koopman and Sportiche, 1991; Chomsky, 1995; Adger, 2003; Citko, 2011). Significantly, these two postulates also provide a particularly useful method for reconstructing the initial evolutionary stage of human grammars, i.e., an intransitive small clause stage, equivalent to the inner VP/SC layer in (1) (Progovac, 2015, 2019). They are also useful in characterizing syntactic variation across languages and constructions, considering this inner layer as the common denominator, or foundation, upon which languages can build further syntactic layers. As such, these postulates are worth keeping and moving forward with.

In conclusion, while there is plenty in Minimalism and predecessors that is empirically and theoretically solid, as well as compatible with gradualist evolution of syntax, this approach would benefit enormously by returning to its more modest but falsifiable claims, aimed at discovering and analyzing the rich landscape of syntactic variation, variability being a key ingredient of selection and evolution.

## Author Contributions

The author confirms being the sole contributor of this work and has approved it for publication.

### Conflict of Interest Statement

The author declares that the research was conducted in the absence of any commercial or financial relationships that could be construed as a potential conflict of interest. The handling editor is currently editing co-organizing a Research Topic with one of the authors LP, and confirms the absence of any other collaboration.

## Footnotes

^1^This is basically the classical characterization of recursion in linguistics, but see e.g. Kinsella (2009), Tomalin (2011), Progovac (2015), who point to immense confusion and inconsistency in the use of the term recursion in syntactic literature; see also Watumull et al. (2014) for offering a clarification of the use of the term, questioning the relevance of embedding. In our eagerness to become highly theoretical (and not easily proven wrong), have we sacrificed the precision in using and defining terms? This is surely one of those aspects that must be fixed.

^2^It is often stated that it does not matter whether languages actually exhibit recursion in this or that construction, or at all, as long as we humans are all cognitively equipped with recursion (e.g. Watumull et al., 2014). This can't be very helpful. If we had never come across a language that showed any recursive capabilities in this sense, would we still maintain that recursion is a crucial property of language?
